# The Role of the N-D1 Linker of the N-Ethylmaleimide-Sensitive Factor in the SNARE Disassembly

**DOI:** 10.1371/journal.pone.0064346

**Published:** 2013-05-07

**Authors:** Cui-Cui Liu, Shan Sun, Sen-Fang Sui

**Affiliations:** State Key Laboratory of Biomembrane and Membrane Biotechnology, Center for Structural Biology, School of Life Sciences, Tsinghua University, Beijing, China; University of South Florida College of Medicine, United States of America

## Abstract

N-ethylmaleimide-sensitive factor (NSF) is a member of the type II AAA+ (ATPase associated with various cellular activities) family. It plays a critical role in intracellular membrane trafficking by disassembling soluble NSF attachment protein receptor (SNARE) complexes. Each NSF protomer consists of an N-terminal domain (N domain) followed by two AAA ATPase domains (D1 and D2) in tandem. The N domain is required for SNARE/α-SNAP binding and the D1 domain accounts for the majority of ATP hydrolysis. Little is known about the role of the N-D1 linker in the NSF function. This study presents detailed mutagenesis analyses of NSF N-D1 linker, dissecting its role in the SNARE disassembly, the SNARE/α-SNAP complex binding, the basal ATPase activity and the SNARE/α-SNAP stimulated ATPase activity. Our results show that the N-terminal region of the N-D1 linker associated mutants cause severe defect in SNARE complex disassembly, but little effects on the SNARE/α-SNAP complex binding, the basal and the SNARE/α-SNAP stimulated ATPase activity, suggesting this region may be involved in the motion transmission from D1 to N domain. Mutating the residues in middle and C-terminal region of the N-D1 linker increases the basal ATPase activity, indicating it may play a role in autoinhibiting NSF activity until it encounters SNARE/α-SNAP complex substrate. Moreover, mutations at the C-terminal sequence GIGG exhibit completely abolished or severely reduced activities of the substrate binding, suggesting that the flexibility of N-D1 linker is critical for the movement of the N domain that is required for the substrate binding. Taken together, these data suggest that the whole N-D1 linker is critical for the biological function of NSF to disassemble SNARE complex substrate with different regions responsible for different roles.

## Introduction

N-ethylmaleimide-sensitive factor (NSF) is one of the first members of AAA+ (ATPase associated with various cellular activities) family and is identified to play a role in vesicular trafficking [1]. The soluble NSF attachment protein receptor (SNARE) proteins are the minimal machinery for membrane fusion [2], and they form parallel, four-helix, coiled-coil SNARE motifs that span the opposing membranes [3]–[5]. The assembly and disassembly of SNARE complex are required for constant vesicular transport. Though other diverse functions for NSF are found [6], [7], the well-confirmed function of NSF is using energy from ATP hydrolysis to disassemble SNARE complex based on its interaction with an adaptor protein, soluble NSF attachment protein (α-SNAP) [8]–[10].

NSF is a ring-shaped homohexameric protein [11], with each protomer consisting of three domains: an N-terminal domain (N domain) and two highly conserved AAA+ domains (D1 and D2 domains) [12], [13]. The N-domain is a positively charged domain in the surface that is important for SNARE/α-SNAP binding [14], [15]. The D1 domain is the major ATPase domain providing the chemical energy by ATP hydrolysis required for the SNARE complex disassembly, while the D2 domain is a degenerate ATPase domain primarily responsible for maintaining NSF as a hexamer [12], [16]. The NSF protein has a relatively very low intrinsic ATPase activity [13], [17]. NSF-N domain has been proposed to exert some control over NSF ATPase activity because antibodies against to it cause a two-fold increase in hydrolytic activity [17], [18]. NSF-D1 domain has a lower affinity for ATP (*K_d_*  = 15–20 μM) and accounts for the majority of basal and SNARE/α-SNAP stimulated ATPase activity [16], [19], [20]. As compared to NSF-D1 domain, NSF-D2 domain has a significantly higher affinity for ATP (*K_d_*  = 30–40 nM) but contributes no significant ATPase activity for NSF [19]. These data demonstrate the importance of the N and D1 domains for NSF ATPase activity and thus for their functions in SNARE complex disassembly.

**Figure 1:**
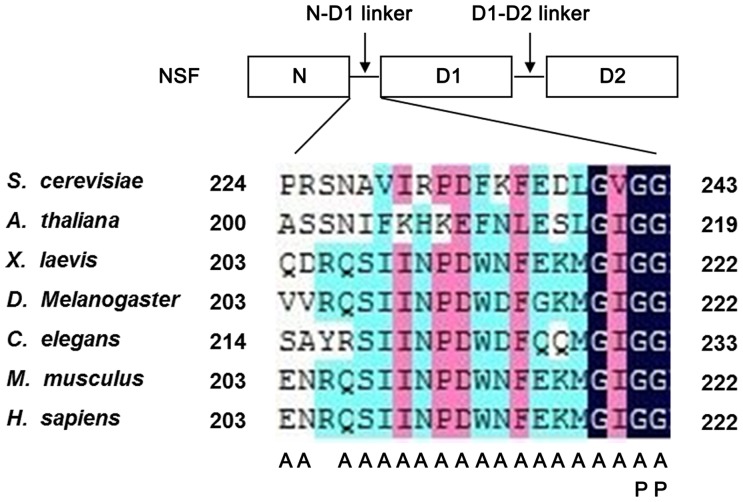
Domain sketch of NSF protein and the amino acid conservation of the N-D1 linker. The diagram shows the structural domains of NSF: the N-terminal domain, D1 and D2 AAA+ modules. The position of the N-D1 linker and D1-D2 linker are indicated. Sequence alignment of the N-D1 linker from different eukaryotic species shows that the N-D1 linker is highly conserved. The numbers at the two ends of the sequence indicate the amino acid residues in NSF. Residues are colored by similarity. The letters under the sequence indicate the amino acid substitutions constructed in this study.

There are two linker regions connecting the N and D1 domains, and the D1 and D2 domains, called the N-D1 linker and the D1-D2 linker, respectively. The NSF N-D1 linker is a highly conserved 20-residue linker, consisting of amino acid residues 203–222 (Fig. 1). Two conserved glycine residues (G221 and G222), present at the C-terminal region of the NSF N-D1 linker, can potentially provide the N-D1 linker with dynamic properties similar to the features seen in the linker of p97 [21], [22]. Structurally the glycine residues are in close proximity to the nucleotide-binding site in p97-D1 domain and could be sensitive to the state of the bound nucleotide [21], [23]. Structural studies of NSF in different nucleotide states showed that the protein undergoes conformation changes during nucleotide binding and hydrolysis, most notably with NSF-N domain changing its disposition relative to the rest of the NSF hexamer [24], [25]. Presumably, communication between the N and D1 domains couples complex assembly/disassembly with the ATP hydrolytic cycle. Despite the functions of the N and D1 domains are clearly demonstrated, little is known about the roles of N-D1 linker for NSF function.

In this study, we examined the role of NSF N-D1 linker using site-directed mutagenesis by monitoring four key activities as follows: the SNARE disassembly, the SNARE/α-SNAP complex binding, the basal ATPase activity and the SNARE/α-SNAP stimulated ATPase activity. Our results suggest that the whole NSF N-D1 linker actually plays a critical role in the biological function of NSF to disassemble SNARE complex with different regions responsible for different roles.

## Results

### Rationale for site-directed mutagenesis of the NSF N-D1 linker

The previous structural comparison and biochemical analysis suggested an important role for the linker between domains in AAA+ proteins [26]–[30]. To investigate the role of the N-D1 linker in NSF, we first compared the sequence similarity of this linker from several different eukaryotic species. As shown in Fig. 1, this linker is highly conserved from yeast to human. Strikingly, the “GIGG” sequence at the C-terminus of the linker is the most highly conserved, implying its functional importance for NSF. Mutating each residue to alanine, which is termed “alanine scanning”, is a useful strategy for identifying functionally important residues in proteins. Additionally, we mutated both glycine residues at the C-terminus to the less flexible proline residues, which might greatly impair the dynamic property of the N-D1 linker.

### N-D1 linker mutants have the same oligomeric state as wild-type NSF

**Figure 2:**
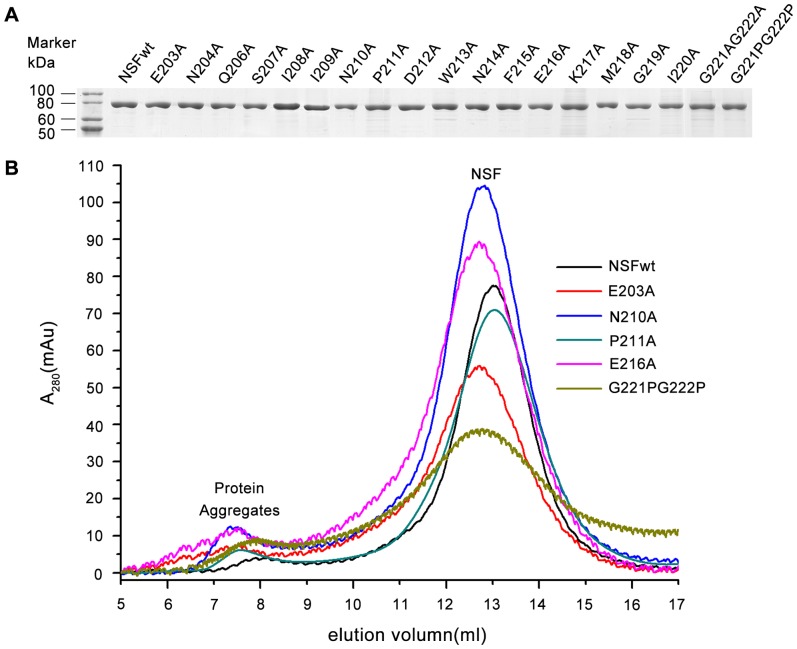
Protein purification and the oligomerization analysis of the NSF mutants. (A) Purity of the NSF proteins. The final purified proteins (3 μg of each proteins) were resolved by 13.5% SDS-PAGE and the gel was stained with Coomassie Brilliant Blue. The molecular size standards are shown on the left. (B) Oligomeric state analysis by size-exclusion chromatography in the buffer containing ADP-AlFx. The typical profiles of NSF (WT), E203A (a representative mutant of N-terminal region), P211A (a representative mutant of middle region), G221PG222P (a representative mutant of C-terminal region), N210A and E216A (the mutants that show the strongest effect in SNARE disassembly assay) from a Superose-6 column were present.

Plasmids expressing wild-type NSF and N-D1 linker mutants were introduced into *E. coli* BL21 (DE3). The proteins were initially purified by Ni-NTA superflow affinity chromatography, then further purified by gel filtration chromatography using a Superdex-200 column. The final purity was analyzed by the SDS-PAGE gel, showing the high purity of each protein (Fig. 2A).

The purified protein was then applied to the Superose-6 column to assess its oligomeric state. All of the mutants showed essentially the same fractionation profile as wild-type NSF, as was evidenced by the typical elution profiles of wild-type NSF and some N-D1 linker mutants shown in Fig. 2B. Excepting the small void volume peak, which contains irreversibly aggregated proteins, the main volume pick is hexameric NSF. This indicates that introduction of the mutations into NSF did not change their oligomeric states.

### The N-terminal region mutants of the NSF N-D1 linker cause significant defects in the disassembly of the SNARE complex

**Figure 3:**
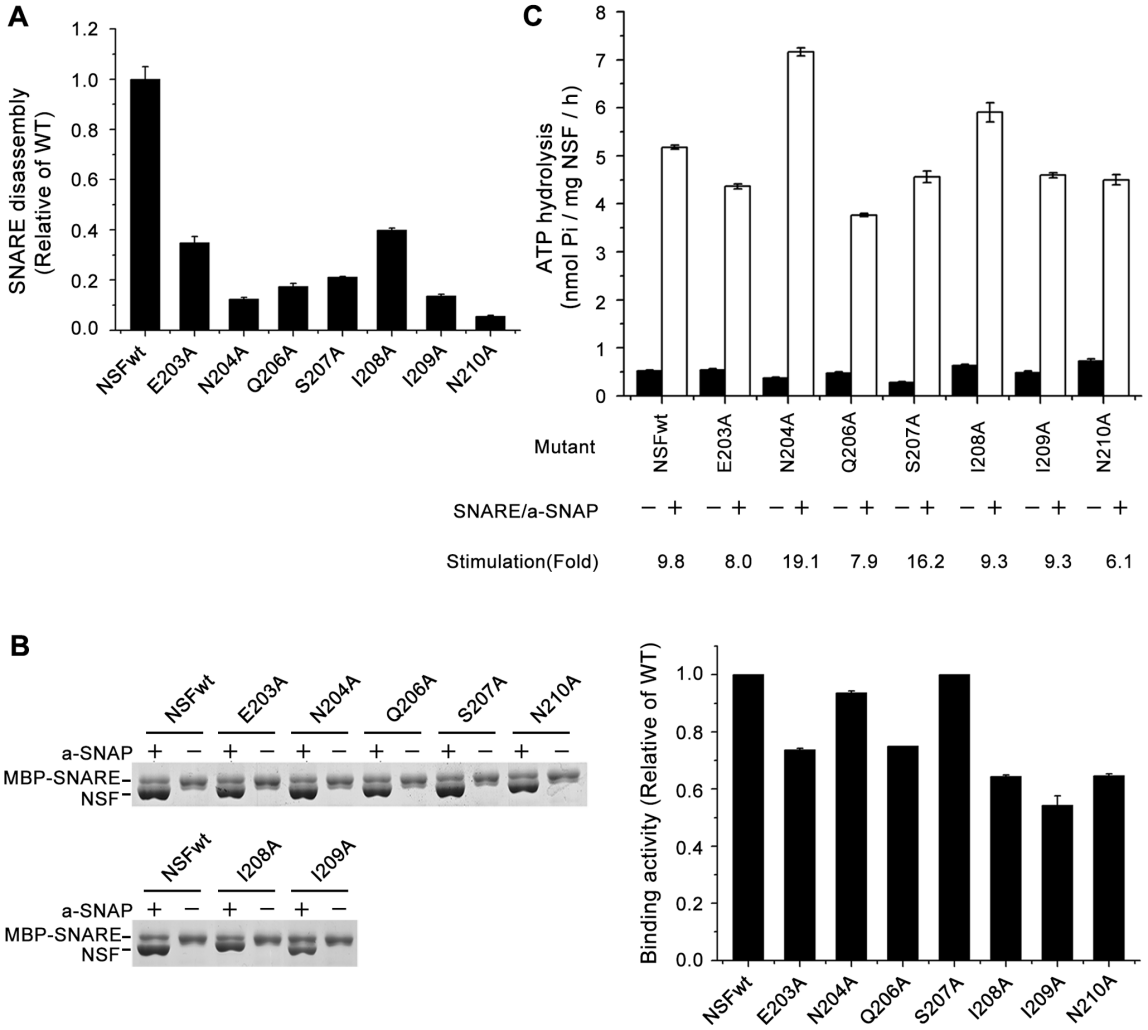
Mutational effects of the N-terminal region of the NSF N-D1 linker. (A) SNARE disassembly by wild-type and mutant NSF. SNARE complexes were incubated with wild-type or mutant NSF, and α-SNAP in the presence of Mg^2+^-ATP at 37°C for 0 min, 20 min and 60 min, followed by the addition of SDS-PAGE loading buffer and analyzed by SDS-PAGE. The SNARE proteins were quantified by densitometry using ImageJ. The histogram shows the SNARE disassembly activities of wild-type and mutated NSF at 60 min averaged from three independent measurements and calculated as follows: disassembled protein, obtained by subtracting remaining protein (60 min) from total protein (0 min), divided by total protein (0 min). Values have been normalized to that of wild-type (WT) NSF. Error bars indicate the standard deviation. (B) Binding of wild-type and mutant NSF to the SNARE/α-SNAP complex. Wild-type or mutant NSF proteins were incubated with MBP-SNARE complexes in the presence (+) or absence (−) of α-SNAP under the ADP-AlFx state. The bound proteins were collected with amylose magnetic beads, eluted with 10 mM maltose and analyzed by SDS-PAGE. The gels (left panel) presented are representative of at least two separate experiments. The bound proteins were quantified by densitometry using ImageJ (right panel). (C) Basal and SNARE/α-SNAP stimulated ATPase activities of wild-type and mutant NSF. Standard ATPase reactions were carried out using 3 μg of wild-type or mutant NSF in the ATPase assay buffer. SNARE/α-SNAP complex was prepared as described in Materials and Methods. Basal and stimulated ATPase activities were measured in the absence (−) or presence (+) of SNARE/α-SNAP complex at 25°C for 1 h, respectively. The histogram shows the rates of ATP hydrolysis averaged from three independent measurements. Error bars indicate standard deviations. The fold increase in ATPase activity (stimulation) was calculated by dividing the stimulated ATPase activity by the basal ATPase activity.

We first assessed the effect of the mutations at the N-terminal region of the N-D1 linker on the biological function of NSF to disassemble SNARE complex. To assay for this disassembly activity, SNARE complexes were preincubated with wild-type NSF or the mutants and α-SNAP on ice, following the addition of 2 mM Mg^2+^-ATP. The reactions were performed at 37°C for the indicated times, then immediately stopped with the addition of the SDS-PAGE loading buffer and analyzed by SDS-PAGE. As expected, the amount of SNARE complexes progressively decreased with the increased incubation time (Fig. S1). But all of the mutants, E203A, N204A, Q206A, S207A, I208A, I209A and N210A, had severe defect. None of them were able to disassemble the SNARE complex to the extent of wild-type NSF (Fig. 3A&S1, Table 1). Among them, the N210A mutant shows the most seriously defective in disassembly efficiency, only 6% of that of wild-type NSF (Fig. 3A, Table 1).

**Table 1:** Summary of SNARE disassembly and ATPase activities of NSF N-D1 linker mutants.

	SNARE Disassembly (WT %)	Basal ATPase Activity (WT %)	Stimulated ATPase Activity (WT %)
NSFwt	1.00±0.050	1.00±0.018	1.00±0.009
E203A	0.35±0.025	1.03±0.020	0.84±0.003
N204A	0.12±0.007	0.71±0.012	1.38±0.006
Q206A	0.17±0.012	0.90±0.028	0.73±0.001
S207A	0.21±0.002	0.53±0.010	0.88±0.016
I208A	0.40±0.009	1.20±0.019	1.14±0.201
I209A	0.14±0.007	0.93±0.020	0.89±0.052
N210A	0.06±0.004	1.39±0.034	0.87±0.014
P211A	0.29±0.004	7.44±0.097	1.13±0.024
D212A	0.46±0.001	5.16±0.160	0.96±0.023
W213A	0.06±0.003	6.08±0.128	0.96±0.021
N214A	0.16±0.004	1.47±0.021	0.70±0.007
F215A	0.48±0.008	3.11±0.122	0.62±0.021
E216A	0.04±0.004	8.10±0.105	0.96±0.024
K217A	0.56±0.003	1.02±0.027	1.03±0.010
M218A	0.51±0.000	11.30±0.173	1.39±0.015
G219A	0.03±0.006	8.20±0.002	0.92±0.022
I220A	0.00±0.010	10.99±0.048	1.18±0.023
G221AG222A	0.00±0.006	4.52±0.170	0.77±0.025
G221PG222P	0.00±0.001	4.61±0.113	0.72±0.022

SNARE disassembly, basal and stimulated ATPase activities of wild-type NSF were set as 1.00. The values for the mutants were normalized to wild-type NSF.

Since the defect in the SNARE disassembly could be due to the defect in the SNARE/α-SNAP binding, we next examined the ability of these mutants to bind to the SNARE/α-SNAP complex in the ADP-AlFx (A transition state analog for the hydrolysis step) state. For this binding assay, equal amounts of wild-type or mutant NSF proteins were preincubated with SNARE complex, with or without α-SNAP, in the presence of ADP-AlFx. The binding complexes were recovered with amylose magnetic beads (MBP-VAMP), and the bound proteins were analyzed by SDS-PAGE as shown in Fig. 3B. These mutations showed either no effects (N204A and S207A) or partially reduced effects (E203A, Q206A, I208A, I209A, and N210A) on binding activity compared with wild-type NSF. This indicates that, when fixed at the ADP-AlFx state, the N-terminal region of N-D1 linker mutants can attain the conformation required for SNARE/α-SNAP complex binding.

Then, the basal and stimulated ATPase activity was investigated. Previous studies demonstrated that NSF has a low basal ATPase activity, which is stimulated 2–10-fold by the binding of SNARE/α-SNAP complex [16], [31], [32]. NSF D1 and N domains account for the majority of the basal and SNARE/α-SNAP stimulated ATPase activity [18], [33]. But we do not know the influence of N-D1 linker in the ATPase activity. In our test condition, wild-type NSF produced inorganic phosphate at a rate of 0.53 nmol/μg/h, and SNARE/α-SNAP complex stimulated a 9.8-fold increase in the ATPase activity (Fig. 3C), which are consistent with the published data [13], [31]. All of the mutations showed no or partial effects on the basal ATPase activities compared with wild-type NSF (Fig. 3C, Table 1), indicating that they can bind ATP. This is in agreement with their normal binding activities with the substrate (Fig. 3B), which requires the protein in the ATP-bound state. But surprisingly, these mutations also exhibit stimulated ATPase activities similar to that of wild type NSF. The apparently observation that all the mutations had normal ATPase activities suggests that the residues at the N-terminal region of N-D1 linker have no significant effect on ATP hydrolysis.

In summary, the mutations at the N-terminal region of N-D1 linker (E203A, N204A, Q206A, S207A, I208A, I209A and N210A) had limited effects on the substrate binding activity and ATPase activity compared with their effects on the SNARE disassembly.

### The middle region mutants of the NSF N-D1 linker have increased basal ATPase activity

To determine the role of the middle region of the N-D1 linker in NSF activity, we prepared the individual mutations encompassing this region by mutating each residue from Pro211 to Met218 to alanine, and then examined the effects of these mutations on the SNARE disassembly, the SNARE/α-SNAP complex binding, the basal and stimulated ATPase activity.

**Figure 4:**
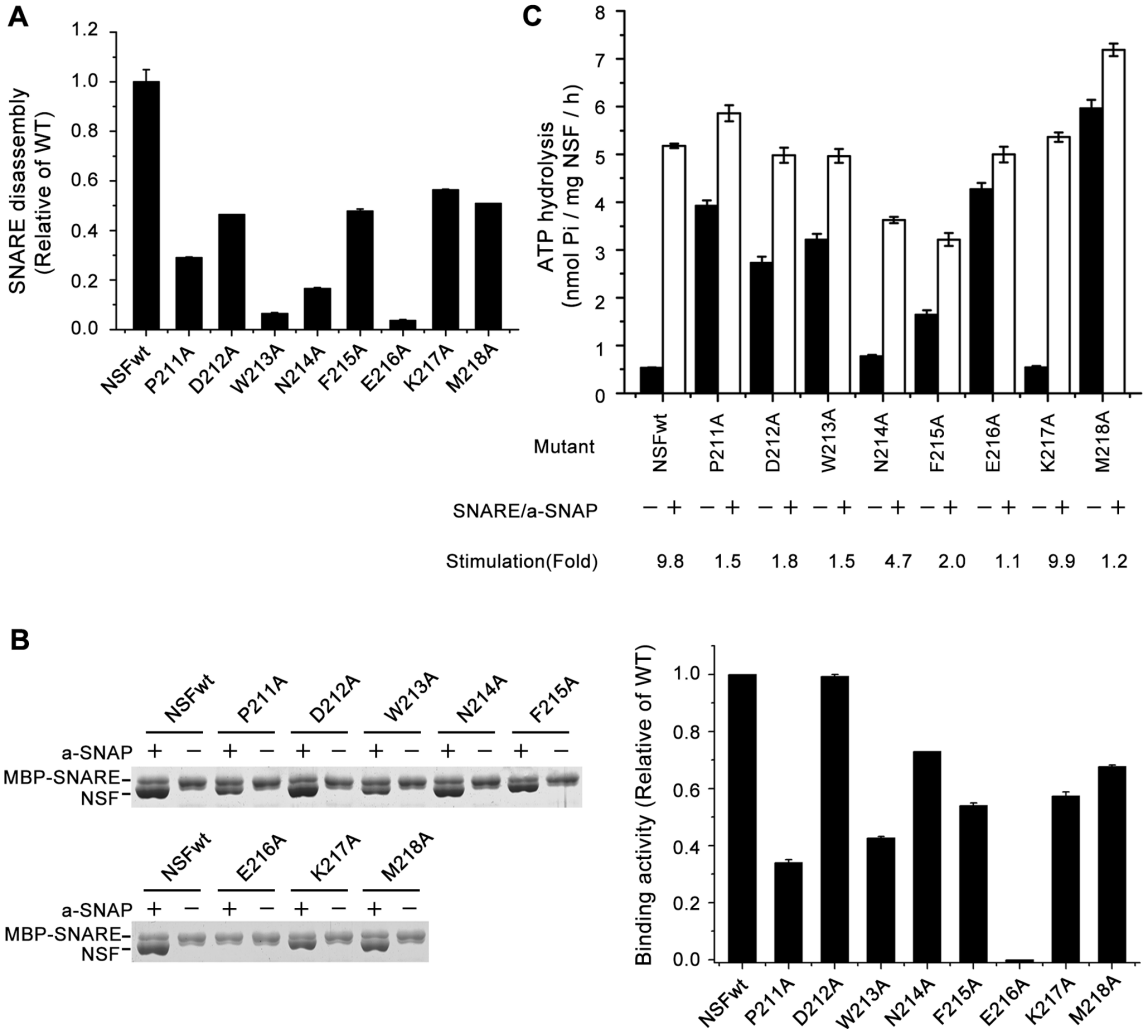
Mutational effects of the middle region of the NSF N-D1 linker. (A) SNARE disassembly by wild-type and mutant NSF proteins. Measurements and presentation of results are the same as in Fig. 3A. (B) Binding of wild-type and mutant NSF proteins to the SNARE/α-SNAP complex. Measurements are the same as in Fig. 3B. (C) Basal and SNARE/α-SNAP stimulated ATPase activities of wild-type and mutant NSF proteins. Measurements and presentation of results are the same as in Fig. 3C.

Similar to the mutations at the N-terminal region, all of the mutants showed reduced abilities to disassemble SNARE (Fig. 4A, Table 1), indicating the middle region is also important for the biological function of NSF. Except for E216A, all other mutants did clearly bind to the SNARE/α-SNAP complex, although to different extents (Fig. 4B). And all the mutants tested have no or marginal effects on the SNARE/α-SNAP stimulated ATPase activity compared with wild-type NSF (Fig. 4C, Table 1). However, all of these mutations result in the elevated basal ATPase activity (Fig. 4C, Table 1). In particular, the P211A, D212A, W213A, F215A, E216A and M218A mutants showed the substantial increase, three times more than wild-type activity (Fig. 4C, Table 1). The basal ATPase activity of the M218A mutant even increased to the extent that is higher than the stimulated ATPase activity of wild-type NSF (Fig. 4C). These results suggest that the residues within the middle region linker may play an important role in helping maintain the conformation of NSF for the low basal ATPase activity.

### The C-terminal region mutants of the NSF N-D1 linker exhibit severe defect in the SNARE/α-SNAP complex binding activity

As demonstrated above, the N-D1 linker of NSF protein is highly conserved from yeast to human (Fig. 1). Especially, the C-terminal region is almost identical with a 4-residue-long GIGG sequence containing a Gly-Gly motif (Fig. 1). From the structural studies of NSF, it is confirmed that the flexible nature of N domain is important for conformational changes of NSF during the ATP hydrolytic cycle [24], [25]. The multiple glycines in the C-terminal region which could impart the conformational flexibility to the N-D1 linker suggest that C-terminal region of the N-D1 linker may modulate the mobility of the NSF-N domain, and affect the NSF function as a consequence. To test this hypothesis, we mutated the residues to Ala, and additionally two adjacent Gly residues to Pro to further decrease the flexibility (Fig. 1).

**Figure 5:**
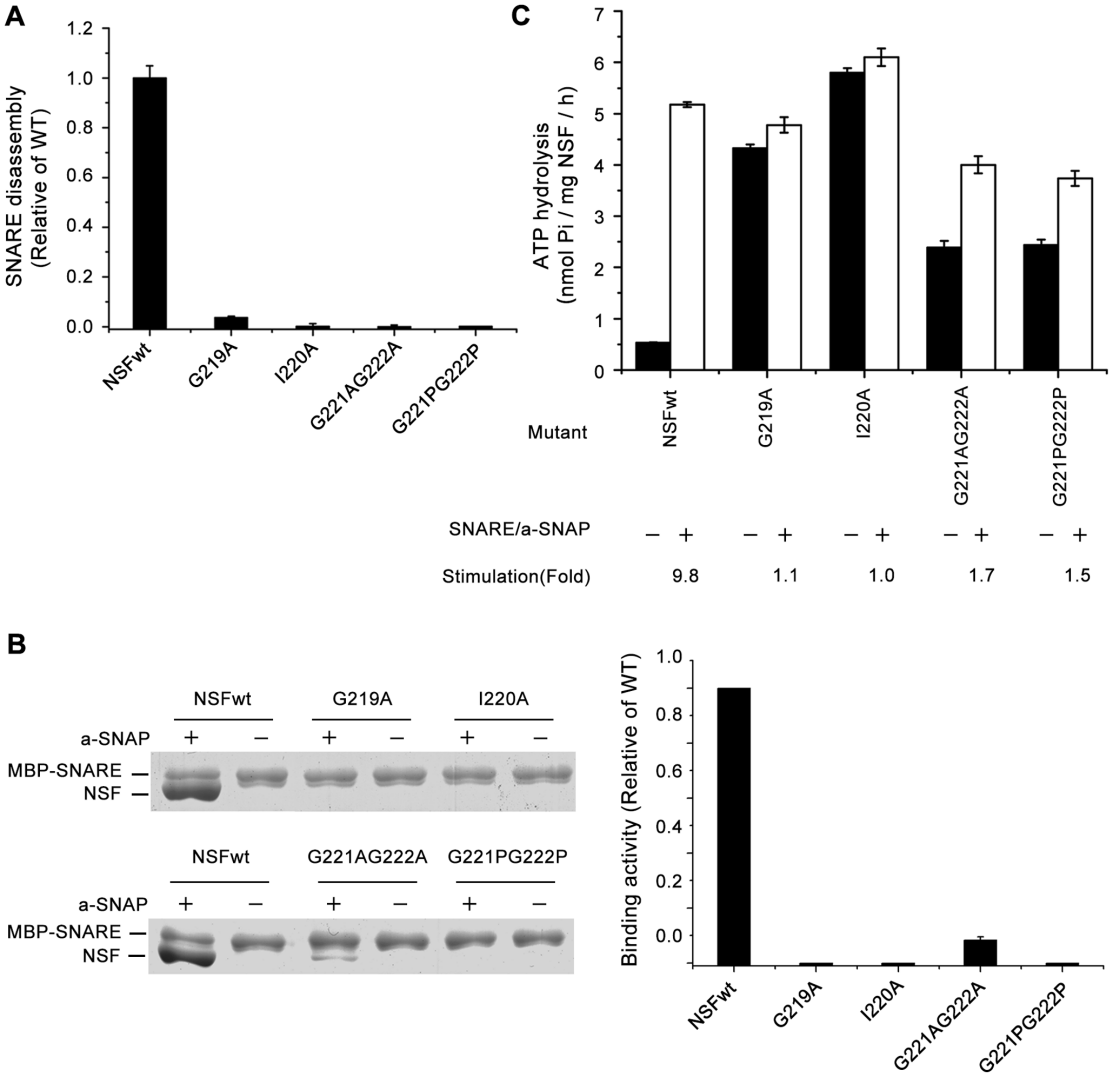
Mutational effects of the C-terminal region of the NSF N-D1 linker. (A) SNARE disassembly by wild-type and mutant NSF proteins. Measurements and presentation of results are the same as in Fig. 3A. (B) Binding of wild-type and mutant NSF proteins to the SNARE/α-SNAP complex. Measurements are the same as in Fig. 3B. (C) Basal and SNARE/α-SNAP stimulated ATPase activities of wild-type and mutant NSF proteins. Measurements and presentation of results are the same as in Fig. 3C.

As shown in Fig. 5A & Table 1, all of the mutations abrogate the ability to disassemble SNARE complex. Similar to the mutations at the middle region of the N-D1 linker, the C-terminal region mutants exhibit an increase in the basal ATPase activity, and marginal impact on the stimulated ATPase activity (Fig. 5C, Table 1). But different from the middle region mutants, these mutants showed severe defect in the SNARE/α-SNAP complex binding activity (Fig. 5B). Three mutants, G219A, I220A, and G221PG222P, fail to bind the substrate, and mutant G221AG222A retains only very weak binding activity compared with wild-type NSF (Fig. 5B), in the ADP-AlFx bound state. This is consistent with our data that the basal ATPase activities of these mutants are similar to their SNARE/α-SNAP stimulated ATPase activities, namely, there are no substrate stimulation effects on these mutants due to their defects in the substrate binding (Fig. 5B&C, Table 1).

## Discussion

Although much research has been conducted since the NSF was first identified [1], the role of the N-D1 linker of NSF is still unclear due to the lack of the structural studies. Here, we present detailed mutagenesis analyses of the N-D1 linker by examining four key activities: the SNARE disassembly, the SNARE/α-SNAP complex binding, the basal ATPase activity and the SNARE/α-SNAP stimulated ATPase activity. Our results suggest that the whole N-D1 linker is critical for the biological function of NSF to disassemble SNARE complex substrate with different regions responsible for different roles.

### The N-terminal region of the NSF N-D1 linker may be involved in the motion transmission from the D1 to the N Domain

Recent structural studies of NSF in different forms of nucleotides by electron microscopy suggested that upon the ATP hydrolysis, the NSF-D1 domain rotated anticlockwise relative to the NSF-D2 domain, which was coupled with the up-to-down movement of the NSF-N domain [24], [25]. Since the NSF-D1 domain is the major ATPase domain [12], the conformational changes of the NSF-D1 domain induced by the ATP hydrolysis must be transmitted to the NSF-N domain. The N and D1 domains of NSF are connected by the highly conserved N-D1 linker which is implied to play an important role in the communication between these two domains despite the lack of experimental evidence. In this study, we present the biochemical evidence to support the hypothesis that the N-D1 linker, especially its N-terminal region, may play a vital role in the motion transmission from the D1 to the N domain.

First, we demonstrated the functional importance of the N-terminal region of the N-D1 linker of NSF by evaluating the mutational effects of the residues in this region on the SNARE disassembly. As shown in Fig. 3A & Table 1, all of the mutations, E203A, N204A, Q206A, S207A, I208A, I209A and N210A, markedly reduced their abilities to disassemble the SNARE complex substrate without exception, indicating the essential role of the N-terminal region of the N-D1 linker in maintaining the functional state of NSF. Second, we examined the activities accounting for the biological function of NSF, including the oligomeric structure, SNARE/α-SNAP complex binding, the basal and the SNARE/α-SNAP stimulated ATPase activity, of these mutants. Surprisingly, these mutants form hexamers (Fig. 2B) and bind to the substrate similar to wild-type NSF (Fig. 3B), and had only limited effects on the basal and the SNARE/α-SNAP stimulated ATPase activity (Fig. 3C, Table 1) compared with their effects on the SNARE disassembly (Fig. 3A, Table 1). Thus, it is reasonable to deduce that the dramatically diminished biological function of these mutants is due to the defect in the motion transmission from the D1 to the N domain, suggesting the N-terminal region of the NSF N-D1 linker may play a key role in the motion transmission process.

Our finding of the NSF N-D1 linker involved in the motion transmission is reminiscent of the previous study of p97, in which the D1-D2 linker mediates the motion transmission from the D2 domain, the major ATPase of p97, to the D1 domain [27], [34]. In this motion-transmission mechanism, the ATP hydrolysis induced motion is transmitted from the D2 to the D1 domain not in the same protomer, but between the interprotomers by the interaction of the D1-D2 linker with the D1 and D2 domains of its neighboring protomer. L464, the key amino acid in the p97 D1-D2 linker, was also located in the N-terminal region of this linker [27]. But different from the p97, most of the key amino acids of NSF identified here are polar and charged amino acids, suggesting that the NSF N-D1 linker transmits the conformational change likely via the polar interactions with the D1 and N domains. Further structural and structure-based mutagenesis analyses will be required to elucidate the detailed mechanism of how the N-D1 linker, especially its N-terminal region, transmits the motion from the D1 to the N domain.

### The middle and C-terminal region of the NSF N-D1 linker may play a role in autoinhibiting NSF ATPase activity until it encounters the SNARE/α-SNAP complex

One striking observation of the mutations in the middle and C-terminal region of the N-D1 linker, except for K217A, is the elevated basal ATPase activity over wild-type NSF, while the SNARE/α-SNAP stimulated ATPase activity is not affected significantly (Fig. 4C, Table 1). It is also notable that some of the mutations exhibit the substantially increased basal ATPase activities similar to or even higher than the SNARE/α-SNAP stimulated ATPase activity of wild-type NSF (Fig. 4C). However, these mutants cannot disassemble the SNARE complex as efficiently as wild-type NSF (Fig. 4A, Table 1). One possible explanation for this is that the high rate of the bound ATP hydrolyzed to ADP and Pi, based on the high basal ATPase activities of the mutants (Fig. 4C, Table 1), cannot maintain these mutants at the stable ATP-bound state, which is required for the SNARE/α-SNAP binding [12]. It should be noted that our substrate binding assay, indicating that these mutants, except for the mutations at E216 and the C-terminal sequence GIGG (see discussion below), can bind to the SNARE/α-SNAP complex (Fig. 4B), was set to the condition in the presence of ADP-AlFx (ATP hydrolysis transition state analog), which physically fixed the mutants at the analogous ATP-bound states. Thus, the results of the substrate binding assay were not conflicted with the above explanation. Another explanation for the defect in SNARE disassembly of the mutants (D212A, N214A, M218A) with the binding activities close to that of wild-type NSF is that the ATPase dependent conformational changes of these mutants are not enough to promote NSF's full activity. Although the absolute values of the stimulated ATPase activities of these mutants are close to that of the wild-type NSF, there are dramatic decreases in their fold increases in ATPase activities (Fig. 4C). This means that the binding of the SNARE/α-SNAP complex to these mutants are unable to stimulate enough ATPase activities to provide the chemical energy to cause large conformational changes required for the SNARE disassembly. Our mutagenesis studies indicate that the ATPase activity of NSF is significantly autoinhibited by the middle and C-terminal region of the N-D1 linker. The binding of the SNARE/α-SNAP complex to NSF releases this autoinhibition, allowing NSF to achieve the enough ATPase activity, generating the chemical energy by ATP hydrolysis to cause large conformational changes in NSF, which is coupled to the disassembly of the SNARE complex [17]. The autoinhibition is also suggested in another AAA+ protein, human VPS4A, where the linker connecting the N domain (MIT domain) and AAA+ domain autoinhibits the ATPase activity of VPS4A until it encounters ESCRT-III substrates [29]. Although such autoinhibition manner is not observed in p97, some of the mutations at the N-D1 linker do increase the ATPase activity [22], [35], which is likely due to the increased flexibility of the N-domain and the N-D1 linker [22], [28]. In the crystal structure of p97, the N-D1 linker, in particular the C-terminal region, is indeed adjacent to the nucleotide-binding pocket [21], [28]. Based on the high similarity of NSF and p97, it is possible that the middle and C-terminal region of the N-D1 linker of NSF may play a role in controlling the ATPase activity of NSF. In the ATP-bound state, this region helps to maintain the structural stability for the low basal activity. Mutations in this region might disrupt this stable conformation, affect the right positioning of the adjacent nucleotide and/or amino acids, and thus led to the increase in the ATPase activity.

### The C-terminal sequence GIGG of the NSF N-D1 linker is essential for the substrate binding

Mutations at the C-terminal sequence GIGG exhibit completely abolished (G219A, I220A, G221PG222P) or severely reduced (G221AG222A) activities of the substrate binding (Fig. 5B). It is well-known that the substrate binding requires NSF to be in its ATP-bound state [12]. Our data show that all of the GIGG mutants do have ATPase activities (Fig. 5C, Table 1), indicating that they can bind to ATP. Thus, their negative effects on the substrate binding are not because they cannot attain the ATP-bound state. Recent structural study shows that the NSF-N domain is flexible, which undergoes the nucleotide-dependent up-to-down movement during the ATP hydrolysis process, namely, it is in an up conformation in the ATP state and in a down conformation in the ADP state [24], [25]. Moreover, the up conformation in the ATP state may be a ready state for the substrate binding [25]. The two adjacent glycine residues are highly conserved in the C-terminus of both N-D1 linker and D1-D2 linker among the AAA+ proteins [21], [22], [27], [34]. Since glycine is the most flexible of the 20-aa residues, these two glycines could serve as a pivot point, allowing the movement of the N-domain [21], [22]. The mutation of both Gly residues to the less flexible residues may constrain the movement of the N-domain, affecting the position of the N-domain in the ATP state, ultimately resulting in the reduced substrate binding activity. Indeed, this hypothesis is supported by the result that the G221PG222P mutant is unable to bind the substrate, while the G221AG222A still retains some weak binding ability (Fig. 5B), since Pro is less flexible than Ala.

## Materials and Methods

### Plasmids and Mutagenesis

Plasmids encoding His_6_-NSF, GST-His_6_-α-SNAP, His_6_-VAMP 1-94 -MBP, His_6_-VAMP 1-94, His_6_-SNAP25 1–90, His_6_-SNAP25 125–206 and GST-Syntaxin 2–253 have been previously described [25]. The site-directed mutants were generated with Easy Mutagenesis System (Transgen) using His_6_-NSF as the template. Eight mutants, P211A, D212A, F215A, E216A, K217A, M218A, G219A, I220A, were cloned into the pQE31 expression vector. Other mutants were cloned into the pET-28a expression vector. All of the mutations were confirmed by DNA sequencing.

### Protein expression and purification

Mutated proteins were expressed in *E. coli* BL21 (DE3) cells at 16°C overnight after addition of 0.3 mM IPTG (isopropyl β-D-thiogalactopyranoside) at an A_600_ of 0.6–0.8. Protein purification was performed as previously described [25], using the Ni-NTA superflow affinity chromatography (Qiagen) followed by the gel filtration chromatography using the Superdex-200 column (GE Healthcare). The purified proteins were analyzed by 13.5% SDS-PAGE gel containing 8 M urea and the gel was stained with Coomassie Blue. Protein quantification was performed by the Bradford method using the bovine serum albumin as the standard.

### Analysis of Oligomeric State of NSF Mutants

The oligomeric analysis of NSF mutants was analyzed by gel filtration chromatography using a Superose-6 column (GE Healthcare) in the buffer containing ADP-AlFx.

### SNARE disassembly assay

SNARE complex was assembled as previously described [25]. For SNARE disassembly assays, SNARE complexes were preincubated with wild-type NSF or the mutants and α-SNAP in 40 μl of a buffer (50 mM HEPES, pH. 7.6, 100 mM NaCl) on ice, following the addition of 2 mM Mg^2+^-ATP. The reactions were performed at 37°C for the indicated times, then aliquots (10 μl) of each reactions were stopped with the addition of the SDS-PAGE loading buffer and immediately loaded onto 13.5% SDS-PAGE gels containing 8 M urea without boiling. The gels were then stained with Coomassie Blue. The SNARE proteins at 0 min and 60 min were quantified by densitometry using ImageJ.

### MBP-SNARE/α-SNAP/NSF binding assay

MBP-SNARE complexes were assembled by mixing Syntaxin, SNAP25-N, SNAP25-C and MBP-VAMP in an equimolar concentration at 4°C overnight. The formed MBP-SNARE complexes were separated from unassembled individual SNAREs by gel filtration using Superdex-200 column (GE Healthcare). For the binding assay, 15 μg of MBP-SNARE complexes were incubated with excess wild-type or mutant NSF proteins (each 100 μg) and α-SNAP (30 μg) for 3 h at 4°C in the binding buffer (50 mM HEPES, pH 7.5, 100 mM NaCl, 1 mM DTT, 2 mM MgCl_2_, and 10% glycerol with 2 mM ADP-AlFx). 40 μl of Amylose Magnetic Beads prewashed with the binding buffer were then added and incubated with rotation. After 4 h incubation at 4°C, the beads were washed three times (1 ml each time) with the binding buffer. The bound protein complexes were eluted with 40 μl of the elution buffer (the binding buffer containing 10 mM maltose) and then analyzed (5 μl of the eluted solution) by 8% SDS-PAGE. The reactions without α-SNAP were used to detect the background of the nonspecific binding. The gels were stained with Coomassie Blue, and the bound proteins were quantified by densitometry using ImageJ.

### ATPase Activity Assay

ATPase assays measure released phosphate based on the formation of colored phosphomolybdate complexes with cationic dye malachite green [36]. Basal ATPase activity assays were carried out using 3 μg of wild-type or mutant NSF proteins in 50 μl of the ATPase assay buffer containing 20 mM HEPES, pH 7.5, 100 mM NaCl, 2 mM MgCl_2_ and 4 mM ATP. The reactions were incubated for 1 h at 25°C, and stopped by adding 20 mM EDTA. Then, 800 μl of the color reagent (0.7 mM malachite green, 8.5 mM ammonium molybdate, 0.1% NP-40, 1.75 M HCl) was added and mixed well. After 5 min incubation at room temperature (25°C), 100 μl of 34% citric acid was added and mixed well. This solution was incubated at room temperature for 15 min, and immediately the absorbance at 645 nm was measured using a spectrophotometer (Amersham). Values were corrected for non-enzymatic breakdown of ATP by running duplicate assays in the absence of NSF protein. The inorganic phosphate released was calculated based on the absorbance standard curve established using KH_2_PO_4_ standards. All assays were repeated at least three times, and the average activities with standard errors of measurement were presented.

To measure the SNARE/α-SNAP-stimulated ATPase activity, SNARE/α-SNAP complexes were assembled for 2 h at 4°C and then incubated with wild-type or mutant NSF proteins in the ATPase assay buffer. The following process was as above. The fold increase in ATPase activity was calculated by dividing the stimulated ATPase activity by the basal ATPase activity.

## Supporting Information

Figure S1 **SNARE disassembly by wild-type and mutant NSF proteins.** SNARE complexes were incubated with wild-type or mutant NSF proteins, and α-SNAP in the presence of 2 mM Mg^2+^-ATP at 37°C for 0 min, 20 min and 60 min, followed by the addition of SDS-PAGE loading buffer and analyzed by SDS-PAGE. The gels were stained with Coomassie Blue.
(TIF)

## References

[1] Block MR, Glick BS, Wilcox CA, Wieland FT, Rothman JE (1988) Purification of an N-ethylmaleimide-sensitive protein catalyzing vesicular transport. Proc Natl Acad Sci U S A 85: 7852–7856. 3186695 10.1073/pnas.85.21.7852 PMC282295

[2] Weber T, Zemelman BV, McNew JA, Westermann B, Gmachl M, et al (1998) SNAREpins: minimal machinery for membrane fusion. Cell 92: 759–772. 9529252 10.1016/s0092-8674(00)81404-x

[3] Stein A, Weber G, Wahl MC, Jahn R (2009) Helical extension of the neuronal SNARE complex into the membrane. Nature 460: 525–528. 19571812 10.1038/nature08156 PMC3108252

[4] Antonin W, Fasshauer D, Becker S, Jahn R, Schneider TR (2002) Crystal structure of the endosomal SNARE complex reveals common structural principles of all SNAREs. Nat Struct Biol 9: 107–111. 11786915 10.1038/nsb746

[5] Sutton RB, Fasshauer D, Jahn R, Brunger AT (1998) Crystal structure of a SNARE complex involved in synaptic exocytosis at 2.4 A resolution. Nature 395: 347–353. 9759724 10.1038/26412

[6] Zhao C, Slevin JT, Whiteheart SW (2007) Cellular functions of NSF: not just SNAPs and SNAREs. FEBS Lett 581: 2140–2149. 17397838 10.1016/j.febslet.2007.03.032 PMC1948069

[7] Whiteheart SW, Matveeva EA (2004) Multiple binding proteins suggest diverse functions for the N-ethylmaleimide sensitive factor. J Struct Biol 146: 32–43. 15037235 10.1016/j.jsb.2003.09.015

[8] Jahn R, Südhof T (1999) Membrane fusion and exocytosis. Annu Rev Biochem 68: 863–911. 10872468 10.1146/annurev.biochem.68.1.863

[9] Söllner T, Whiteheart SW, Brunner M, Erdjument-Bromage H, Geromanos S, et al (1993) SNAP receptors implicated in vesicle targeting and fusion. Nature 362: 318–324. 8455717 10.1038/362318a0

[10] Clary DO, Griff IC, Rothman JE (1990) SNAPs, a family of NSF attachment proteins involved in intracellular membrane fusion in animals and yeast. Cell 61: 709–721. 2111733 10.1016/0092-8674(90)90482-t

[11] Fleming KG, Hohl TM, Yu RC, Muller SA, Wolpensinger B, et al (1998) A revised model for the oligomeric state of the N-ethylmaleimide-sensitive fusion protein, NSF. The Journal of biological chemistry 273: 15675–15681. 9624162 10.1074/jbc.273.25.15675

[12] Nagiec EE, Bernstein A, Whiteheart SW (1995) Each domain of the N-ethylmaleimide-sensitive fusion protein contributes to its transport activity. The Journal of biological chemistry 270: 29182–29188. 7493945 10.1074/jbc.270.49.29182

[13] Tagaya M, Wilson DW, Brunner M, Arango N, Rothman JE (1993) Domain structure of an N-ethylmaleimide-sensitive fusion protein involved in vesicular transport. The Journal of biological chemistry 268: 2662. 8428942

[14] Zhao C, Matveeva EA, Ren Q, Whiteheart SW (2010) Dissecting the N-ethylmaleimide-sensitive factor: required elements of the N and D1 domains. The Journal of biological chemistry 285: 761–772. 19887446 10.1074/jbc.M109.056739 PMC2804225

[15] Yu RC, Jahn R, Brunger AT (1999) NSF N-terminal domain crystal structure: models of NSF function. Mol Cell 4: 97–107. 10445031 10.1016/s1097-2765(00)80191-4

[16] Steel GJ, Morgan A (1998) Selective stimulation of the D1 ATPase domain of N-ethylmaleimide-sensitive fusion protein (NSF) by soluble NSF attachment proteins. FEBS Lett 423: 113–116. 9506852 10.1016/s0014-5793(98)00072-6

[17] Zhao C, Smith EC, Whiteheart SW (2012) Requirements for the catalytic cycle of the N-ethylmaleimide-Sensitive Factor (NSF). Biochim Biophys Acta 1823: 159–171. 21689688 10.1016/j.bbamcr.2011.06.003 PMC3983028

[18] Sumida M, Hong RM, Tagaya M (1994) Role of two nucleotide-binding regions in an N-ethylmaleimide-sensitive factor involved in vesicle-mediated protein transport. The Journal of biological chemistry 269: 20636–20641. 8051162

[19] Matveeva EA, He P, Whiteheart SW (1997) N-Ethylmaleimide-sensitive fusion protein contains high and low affinity ATP-binding sites that are functionally distinct. The Journal of biological chemistry 272: 26413–26418. 9334216 10.1074/jbc.272.42.26413

[20] Whiteheart SW, Rossnagel K, Buhrow SA, Brunner M, Jaenicke R, et al (1994) N-ethylmaleimide-sensitive fusion protein: a trimeric ATPase whose hydrolysis of ATP is required for membrane fusion. J Cell Biol 126: 945–954. 8051214 10.1083/jcb.126.4.945 PMC2120109

[21] Zhang X, Shaw A, Bates PA, Newman RH, Gowen B, et al (2000) Structure of the AAA ATPase p97. Mol Cell 6: 1473–1484. 11163219 10.1016/s1097-2765(00)00143-x

[22] Niwa H, Ewens CA, Tsang C, Yeung HO, Zhang X, et al (2012) The role of the N-domain in the ATPase activity of the mammalian AAA ATPase p97/VCP. The Journal of biological chemistry 287: 8561–8570. 22270372 10.1074/jbc.M111.302778 PMC3318706

[23] May AP, Whiteheart SW, Weis WI (2001) Unraveling the mechanism of the vesicle transport ATPase NSF, the N-ethylmaleimide-sensitive factor. The Journal of biological chemistry 276: 21991–21994. 11301340 10.1074/jbc.R100013200

[24] Moeller A, Zhao C, Fried MG, Wilson-Kubalek EM, Carragher B, et al (2012) Nucleotide-dependent conformational changes in the N-Ethylmaleimide Sensitive Factor (NSF) and their potential role in SNARE complex disassembly. J Struct Biol 177: 335–343. 22245547 10.1016/j.jsb.2011.12.018 PMC3382979

[25] Chang LF, Chen S, Liu CC, Pan X, Jiang J, et al (2012) Structural characterization of full-length NSF and 20S particles. Nat Struct Mol Biol 19: 268–275. 22307055 10.1038/nsmb.2237

[26] Zhang T, Ploetz EA, Nagy M, Doyle SM, Wickner S, et al (2012) Flexible connection of the N-terminal domain in ClpB modulates substrate binding and the aggregate reactivation efficiency. Proteins 80: 2758–2768. 22890624 10.1002/prot.24159 PMC3486956

[27] Li G, Huang C, Zhao G, Lennarz WJ (2012) Interprotomer motion-transmission mechanism for the hexameric AAA ATPase p97. Proc Natl Acad Sci U S A 109: 3737–3741. 22355145 10.1073/pnas.1200255109 PMC3309748

[28] Tang WK, Li D, Li C-c, Esser L, Dai R, et al (2010) A novel ATP-dependent conformation in p97 N-D1 fragment revealed by crystal structures of disease-related mutants. EMBO J 29: 2217–2229. 20512113 10.1038/emboj.2010.104 PMC2905243

[29] Merrill SA, Hanson PI (2010) Activation of human VPS4A by ESCRT-III proteins reveals ability of substrates to relieve enzyme autoinhibition. The Journal of biological chemistry 285: 35428–35438. 20805225 10.1074/jbc.M110.126318 PMC2975166

[30] Smith GR, Contreras-Moreira B, Zhang X, Bates PA (2004) A link between sequence conservation and domain motion within the AAA+ family. J Struct Biol 146: 189–204. 15037250 10.1016/j.jsb.2003.11.022

[31] Matveeva E, Whiteheart SW (1998) The effects of SNAP/SNARE complexes on the ATPase of NSF. FEBS Lett 435: 211–214. 9762911 10.1016/s0014-5793(98)01071-0

[32] Morgan A, Dimaline R, Burgoyne RD (1994) The ATPase activity of N-ethylmaleimide-sensitive fusion protein (NSF) is regulated by soluble NSF attachment proteins. The Journal of biological chemistry 269: 29347–29350. 7961908

[33] Matveeva EA, May AP, He P, Whiteheart SW (2002) Uncoupling the ATPase activity of the N-ethylmaleimide sensitive factor (NSF) from 20S complex disassembly. Biochemistry 41: 530–536. 11781091 10.1021/bi015632s

[34] Huang C, Li G, Lennarz WJ (2012) Dynamic flexibility of the ATPase p97 is important for its interprotomer motion transmission. Proc Natl Acad Sci U S A 109: 9792–9797. 22675116 10.1073/pnas.1205853109 PMC3382474

[35] Manno A, Noguchi M, Fukushi J, Motohashi Y, Kakizuka A (2010) Enhanced ATPase activities as a primary defect of mutant valosin-containing proteins that cause inclusion body myopathy associated with Paget disease of bone and frontotemporal dementia. Genes Cells 15: 911–922. 20604808 10.1111/j.1365-2443.2010.01428.x

[36] Lanzetta PA, Alvarez LJ, Reinach PS, Candia OA (1979) An improved assay for nanomole amounts of inorganic phosphate. Analytical biochemistry 100: 95–97. 161695 10.1016/0003-2697(79)90115-5

