# Supplementary figures and images for: The Role of the N-D1 Linker of the N-Ethylmaleimide-Sensitive Factor in the SNARE Disassembly
Source: PLoS One. 2013 May 7;8(5):e64346. doi: 10.1371/journal.pone.0064346 (PMC3646813; doi:10.1371/journal.pone.0064346)

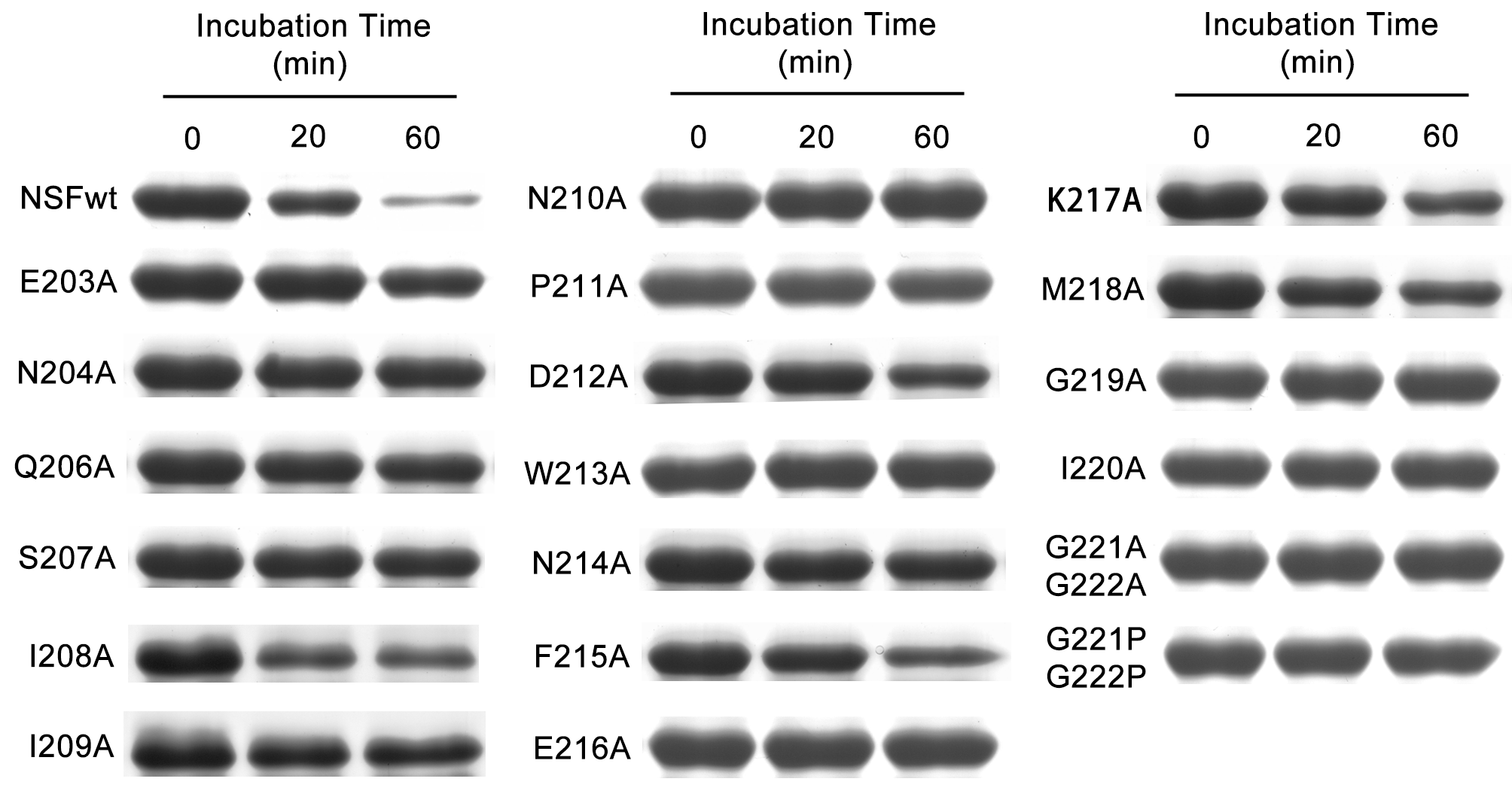

Supplement: Figure S1 — SNARE disassembly by wild-type and mutant NSF proteins. SNARE complexes were incubated with wild-type or mutant NSF proteins, and α-SNAP in the presence of 2 mM Mg2+-ATP at 37°C for 0 min, 20 min and 60 min, followed by the addition of SDS-PAGE loading buffer and analyzed by SDS-PAGE. The gels were stained with Coomassie Blue. (TIF) [file pone.0064346.s001.tif]
